# Symptoms of major depressive disorder and post-traumatic stress disorder in veterans with mild traumatic brain injury: A network analysis

**DOI:** 10.1371/journal.pone.0283101

**Published:** 2023-05-04

**Authors:** Shuyuan Shi, Erin Almklov, Niloofar Afari, James O. E. Pittman

**Affiliations:** 1 Department of Psychology, University of British Columbia, Vancouver, BC, Canada; 2 VA Center of Excellence for Stress and Mental Health, San Diego, CA, United States of America; 3 VA San Diego Healthcare System, San Diego, CA, United States of America; 4 Department of Psychiatry, University of California San Diego, La Jolla, CA, United States of America; Penn State Health, UNITED STATES

## Abstract

Mild Traumatic Brain Injury (mTBI, or concussion) is a debilitating condition that often leads to persistent cognitive and mental health problems post-injury. Post-traumatic Stress Disorder (PTSD) and Major Depressive Disorder (MDD) are two most commonly occurring mental health problems following mTBI and are suggested to be strong contributors to the persistent post-concussion symptoms. Thus, it is important to understand the symptomatology of PTSD and MDD post-mTBI, to better inform targets for behavioral health interventions. Therefore, the current study examined the symptom structure of post-mTBI co-morbid PTSD and MDD through network approaches; we compared the network structure of participants with a positive mTBI screen (N = 753) to the network structure of participants with a negative mTBI screen (N = 2044); lastly, we examined a network of PTSD and MDD symptoms with clinical covariates in a positive mTBI sample. We found that *feeling distant/cutoff* (P10) and *difficulty concentrating* (P15) were the most central symptoms in the positive mTBI network and sleep problems were the most prominent bridge nodes across the disorders. No significant difference between the positive and negative mTBI network were found through network comparison tests. Moreover, anxiety and insomnia were strongly associated with sleep symptoms and irritability symptoms, and emotional support and resilience were potential buffers against most of the PTSD and MDD symptoms. The results of this study might be particularly useful for identifying targets (i.e., *feeling distant*, concentration and sleep problems) for screening, monitoring and treatment after concussion to better inform post-mTBI mental health care and to improve treatment outcomes.

## Introduction

Mild Traumatic Brain Injury (mTBI) is a common injury and a leading cause of disability in the United States [1]. Although most patients with mTBI recover within three months following injury, up to one third of patients develop persistent post-concussion symptoms [2–6]. Moreover, patients with mTBI are more likely to develop debilitating mental health complications, especially post-traumatic stress disorder (PTSD) and major depressive disorder (MDD), which in turn can lead to poorer recovery and persistent post-concussion symptoms [7–12]. The population-based prevalence rate of mTBI is estimated at 0.6% among general adult population [13]; while for military veterans, a highly combat-exposed population, prevalence rates are much higher (i.e., 12–23%) [14–16]. Veterans also report higher rates of mental health complications following injury [15]. Of the approximately 25,000 post-deployment veterans returning from Afghanistan or Iraq between 2009 and 2014, a significant proportion (10%-30%) of those who received a positive mTBI diagnosis had symptoms that persisted for more than 3 months post-injury [17], and one of the most salient features of nonrecovery from mTBI are post-mTBI mental health problems [18]. Among patients with mTBI, up to 39% of veterans report PTSD [19], and 50% of veterans report MDD [20], compared to around 18% civilians report PTSD and/or depression [21]. Therefore, it is important to better understand the symptom structures of commonly occurring post-mTBI mental health problems and to develop tailored post-injury assessments and interventions for the veteran population.

PTSD and MDD are among the most common mental health disorders occurring post-injury among veterans [16, 22]. It is reported that among patients with PTSD, 55% have also been diagnosed with MDD at least once in their lifetime [23]. Both MDD and PTSD are associated with impaired physical functioning [24, 25], greater healthcare utilization and costs [26], and higher rates of disability [27]. Patients with co-morbid PTSD and MDD have also reported poorer treatment outcomes, increased health burden and increased suicidal behaviors compared to patients with only PTSD or MDD [28, 29]. Furthermore, one study found that among nearly 120,000 combat veterans, co-morbid PTSD and MDD post-mTBI is associated with elevated levels of chronic pain and pain-related disability [30]. Proactive management of mental health complications may improve overall recovery and return to productivity after mTBI [31]. However, to our knowledge, there lack systematic screening and proactive management for PTSD and MDD following mTBI in the veteran population. Understanding the symptom structure of comorbid PTSD and MDD in the context of mTBI might inform targets for proactive screening and treatment for the military population.

PTSD and MDD share a number of criterion symptoms, as listed in the most recent Diagnostic and Statistical Manual of Mental Disorders [32]. Multiple theories have been raised trying to explain the high prevalence of the comorbidity between PTSD and MDD. Factor analysis studies suggest that PTSD/MDD comorbidity is due to one or more shared underlying dimensions [33, 34], while other studies suggest that such comorbidity is caused by dimensional communality—items/dimensions are correlated but disorders are distinct [35]. However, in these above theories, symptoms are viewed as indicators of latent variable(s) that represent the disorders, and none have taken the symptom-level associations into account.

Recently, a network approach for understanding the symptomatology of comorbid PTSD and MDD has gained increasing attention. The network approaches holds that symptom level associations constitute the disorder and comorbidity [36–38]. Unlike previous categorical or dimensional perspectives which view symptoms as equal contributors to one underlying variable, network perspective assumes interactive and causal relationships between symptoms that can trigger and/or reinforce each other, which constitute a dynamic network. According to the network theory, a symptom is central in the network when it triggers most activation of other symptoms [39], and a bridging symptom is a symptom that is central in connecting the two disorders, which might be driving comorbidity [40]. Network analysis is still a relatively new method in studying the complex symptomatology both between and within disorders.

Over the past few years, many studies have utilized this novel approach to investigate the symptom structure of PTSD or MDD [41–43], while few have examined the symptom structure of co-occurring PTSD/MDD, despite the high comorbidity rate [33]. Findings from the limited studies that have investigated the comorbid PTSD/MDD symptom structure network among various populations have identified sleep problems, concentration difficulties, irritability and anhedonia to be central symptoms that trigger most activation of other symptoms across the network [44–47]. Afzali et al. (2017) found that bridging symptoms are not limited to the overlapping criterion symptoms outlined in DSM-V; they identified five non-overlapping symptoms (i.e., sense of foreshortened future, feelings of guilt, feeling sad, psychomotor retardation and flashbacks) that are central in the network and can spread activation to other symptoms which result in an expression of the disorder [47]. However, studies that have investigated comorbidity are scarce and are often limited in sample size or target population, and no extant literature has examined the comorbid symptom structure of PTSD and MDD within a mTBI population.

To bridge the knowledge gap, the current study aimed to: 1) establish a network of comorbid PTSD and MDD symptoms, and identify the central symptoms that might be contributing to persistent post-concussive symptoms in the mTBI sample; 2) compare the network of co-morbid PTSD and MDD symptoms among veterans with and without mTBI; and 3) include clinical covariates (i.e., anxiety, insomnia, resilience and emotional support) into the comorbidity network to examine whether they display particular relationships with certain symptoms. The findings of this investigation may have important clinical implications, such as better informing symptom targets for assessment, treatment, and monitoring of post-MTBI patients to prevent the development of chronic health conditions.

## Materials & method

### Study sample

This study was a retrospective cross-sectional evaluation of 2,797 veterans registering for care at the VA San Diego Healthcare System (VASDHS) between July 1, 2014, and November 22, 2017. Data were collected as part of standard clinical screening processes in Transition Care Management (TCM) clinics with the use of eScreening, an electronic mobile self-report screening tool [48]. Participants were primarily male (84.7%) with a mean age of 36.3 (*SD* = 9.0).

### Measures

#### Sociodemographic and service history

A researcher designed self-report questionnaire was used to record age, gender, race/ethnicity, relationship status, and work status. Service history related to branch of service, number of deployments, and combat exposure were also captured with the questionnaire.

#### MDD symptoms

Patient Health Questionnaire 9-Item Depression Module (PHQ-9) [49] measured depression symptoms occurring within the past two week period. Items are rated on a 4-point scale with a maximum score of 27. Higher scores indicate greater severity, with the clinically cutoff score of 10 and above indicating moderate to severe depressive symptoms. The PHQ-9 is generally a reliable and valid measure of depression, although evidence for inter-rater reliability is lacking [49, 50]. The questionnaire items are presented in Table 1.

**Table 1:** PCL-C and PHQ-9 items.

Item	PHQ-9	PCL-C
**1**	Little interest or pleasure in doing things	Repeated disturbing memories, thoughts or images of a stressful experience from the past
**2**	Feeling down, depressed, or hopeless	Repeated disturbing dreams of a stressful experience from the past
**3**	Trouble falling/staying asleep, sleeping too much	Suddenly acting or feeling as if a stressful experience were happening again (as if you were reliving it)
**4**	Feeling tired or having little energy	Feeling very upset when something reminded you of a stressful experience from the past
**5**	Poor appetite or overeating	Having physical reactions (e.g., heart pounding, trouble breathing, sweating) when something reminded you of a stressful experience from the past
**6**	Feeling bad about yourself or that you are a failure or have let yourself or your family down	Avoiding memories, thoughts, or feelings related to the stressful experience
**7**	Trouble concentrating on things, such as reading the newspaper or watching television	Avoiding activities or situations because they reminded you of a stressful experience from the past
**8**	Moving or speaking slowly that other people could have noticed; or being so fidgety or restless that you have been moving around a lot than usual	Trouble remembering important parts of a stressful experience from the past
**9**	Thoughts that you would be better off dead or of hurting yourself in some way	Loss of interest in activities that you used to enjoy
**10**		Feeling distant or cutoff from other people
**11**		Feeling emotionally numb or being unable to have loving feelings for those close to you
**12**		Feeling as if your future will be cut short
**13**		Trouble falling or staying asleep
**14**		Feeling irritable or having angry outbursts
**15**		Having difficulty concentrating
**16**		Being “super-alert” or watchful or on guard
**17**		Feeling jumpy or easily startled

#### PTSD symptoms

The seventeen item PTSD Checklist—Civilian Version (PCL-C) [51] was used to assess PTSD symptoms over the past month. The items were scored on a 5-point scale with scores range from 17–85, such that higher scores indicating greater severity. The clinically cutoff score of 30 and above indicating moderate to severe PTSD symptoms The PCL-C was chosen for its high internal consistency across military and nonclinical populations [51]. The questionnaire items are presented in Table 1.

#### MTBI screening

The Brief Traumatic Brain Injury Screen (BTBIS) [52] is a three item questionnaire used to detect mTBI following combat deployment. It has been routinely administered within military samples to screen for potential mTBIs. A participant is considered to have a positive screen if he or she selects items on the first two questions, which asks for mTBI exposures during their deployment and altered mental status associated with the injury.

#### Clinical covariates

Anxiety was assessed with the seven item Generalized Anxiety Disorder 7 scale (GAD-7) [53]. Sleep symptoms present within the past two week period were measured with the seven item Insomnia Severity Index (ISI) [54]. Resilience was assessed with the 10-item Connor-Davidson Resilience Scale (CD-RISC 10) [55]. Emotional support was measured by self-reported scores on the Patient-Reported Outcomes Measurement Information System (PROMIS) Emotional Support questionnaire [56]. The validity and reliability of above measures were proved by previous literature.

### Analysis plan

#### Assessment of psychiatric symptoms

Descriptive statistics were used to characterize the study sample. Chi-square tests were used to compare the rates of PTSD, MDD, and comorbidity across mTBI and non-mTBI samples. Furthermore, independent t-tests were conducted to compare the PTSD total score and MDD total score between mTBI and non-mTBI samples, in order to assess if one group report significantly worse symptoms than the other group.

#### Network analysis

*Network visualization*. Network analyses of co-morbid PTSD/MDD symptom structures across mTBI and non-mTBI sample were conducted in R using the *qgraph* package [57]. PTSD symptoms, MDD symptoms were included in the network, with each symptom represented by a node and relationship between symptoms represented by edges. The strength of the relationship was represented by line (edge) thickness, with blue lines indicating positive correlations and orange lines indicating negative correlations. To control for false positive rates, we applied the Least Absolute Shrinkage and Selector Operator (LASSO) regularization procedure which sets all weak partial correlations (determined by a set parameter) to exact zero. The parameter is chosen using the Extended Bayesian Information Criterion (EBIC), and is set as 0.5 as recommended by previous research [58].

*Node centrality measures*. The relative importance of the symptoms within the networks were assessed through the centrality function in the package, and node strength, betweenness, closeness and expected influence (EI) was calculated for each symptom [59] to indicate node centrality. Specifically, we chose EI to indicate node (symptom) centrality, as previous research (which the current study came to the same conclusion through centrality stability tests, see Fig 3) consistently considered EI to be a more reliable and stable measure of centrality than measures like closeness and betweenness [39, 60, 61]. EI is calculated by summing the weight of all positive and negative edges of a node, and higher EI values indicate greater centrality/importance of the node/symptom to the network/disorder. Moreover, EI was computed for bridge nodes–nodes that have symptoms level connections with nodes of the other disorder—for all networks [40]. Tests of differences were also conducted to better distinguish significant differences in edge weights and node EI.

*Network robustness (accuracy and stability)*. Network accuracy was assessed using the *bootnet* package [59]. Through a non-parametric approach, we bootstrapped 95% confidence intervals of the edge weights 1000 times to test the interrelations’ accuracy. Network stability was scrutinized through re-calculating the correlation stability coefficients (CS-coefficient) using subsetting bootstrap. The network is identified as stable if the interrelatedness coefficients are similar across the subsets. A CS-coefficient that is greater 0.70 indicates good network stability [59].

*Network comparison*. To compare the two networks, correlation test and network structure invariance tests were ran [62]. Moreover, the global network strength (i.e., the overall network connectivity calculated by the sum of absolute edge weight values) and global network expected influence (i.e., the degree to which the symptoms are assumed to enhance each other by the sum of all positive and negative edge weight values) of the two networks was compared using the R package *NCT*.

*Clinical covariates*. A new network model with comorbid PTSD and MDD symptoms and clinical covariates was established for the positive mTBI group. The node centrality test, network accuracy test, as well as network stability test was conducted to see if clinical covariates were important influential factors for the development/progression of the post-mTBI comorbid symptoms.

## Results

### Descriptive results

Veterans’ self-reported sociodemographic and service history characteristics are provided in Table 2. This sample of veterans consist of mostly male (84.7%) with a mean age of 36.3 (*SD* = 9.0). Around twenty-six percent of the sample identified as Hispanic/Latino. Majority of the sample were Caucasian (59.8%) followed by African American (15.6%) and Asian (13.9%). Over eighty percent of the sample had completed some college or beyond, and approximately 58% reported being unemployed while 31% had full-time employment with varied levels of income. Consistent with local demographics, 93.8% had served active duty mostly in the Navy (48.8%) or Marines (32.1%).

**Table 2:** Demographic of veteran cohorts (N = 2797).

Characteristic	N (%)	Characteristic	N (%)
**Age**	M = 36.3, SD = 9.0	**Employment Status**	
**Gender**		Unemployed	1677 (58.0)
Male	2318 (84.7)	Full time	895 (31.0)
Female	418 (15.3)	Part time	285 (9.9)
**Race**		Seasonal/day labor	33 (1.1)
African American	403 (15.6)	**Income (k = thousands)**	
Asian	358 (13.9)	15k~30k	390 (13.8)
Caucasian	1540 (59.8)	30k~45k	625 (22.1)
Multi-Race	205 (8.0)	45k~60k	520 (18.4)
Pacific Islander	31 (1.2)	60k~75k	391 (13.8)
Native	40 (1.6)	75k~100k	321 (11.4)
**Ethnicity**		Less than 15k	268 (9.5)
Hispanic	679 (25.6)	More than 100k	310 (11.0)
Non-Hispanic	1978 (74.4)	**Service Type**	
**Education**		Active duty	2704 (93.8)
Some high school	17 (0.6)	Reserve	130 (4.5)
GED	38 (1.3)	National guard	50 (1.7)
High school diploma	524 (18.1)	**Service Branch**	
Some college	1271 (43.8)	Navy	1405 (48.8)
Associates degree	339 (11.7)	Marines	924 (32.1)
4-year college degree	484 (16.7)	Army	396 (13.7)
Master’s degree	201 (6.9)	Air force	114 (4.0)
Doctoral degree	26 (0.9)	National guard	30 (1.0)
		Coast guard	13 (0.5)

Chi-square tests revealed that veterans with positive mTBI screening are significantly more likely to develop PTSD, MDD and comorbid MDD/PTSD (as shown in S1 Table in S1 File). Among those who met clinical cutoffs for PTSD and MDD, veterans with positive mTBI report greater MDD and PTSD symptoms severity (*t*(986) = 8.62, *P <* 0.001; *t*(986) = 3.40, *P <* 0.001) than veterans without mTBI.

### Network results

#### mTBI network & non-mTBI network

**Fig 1:**
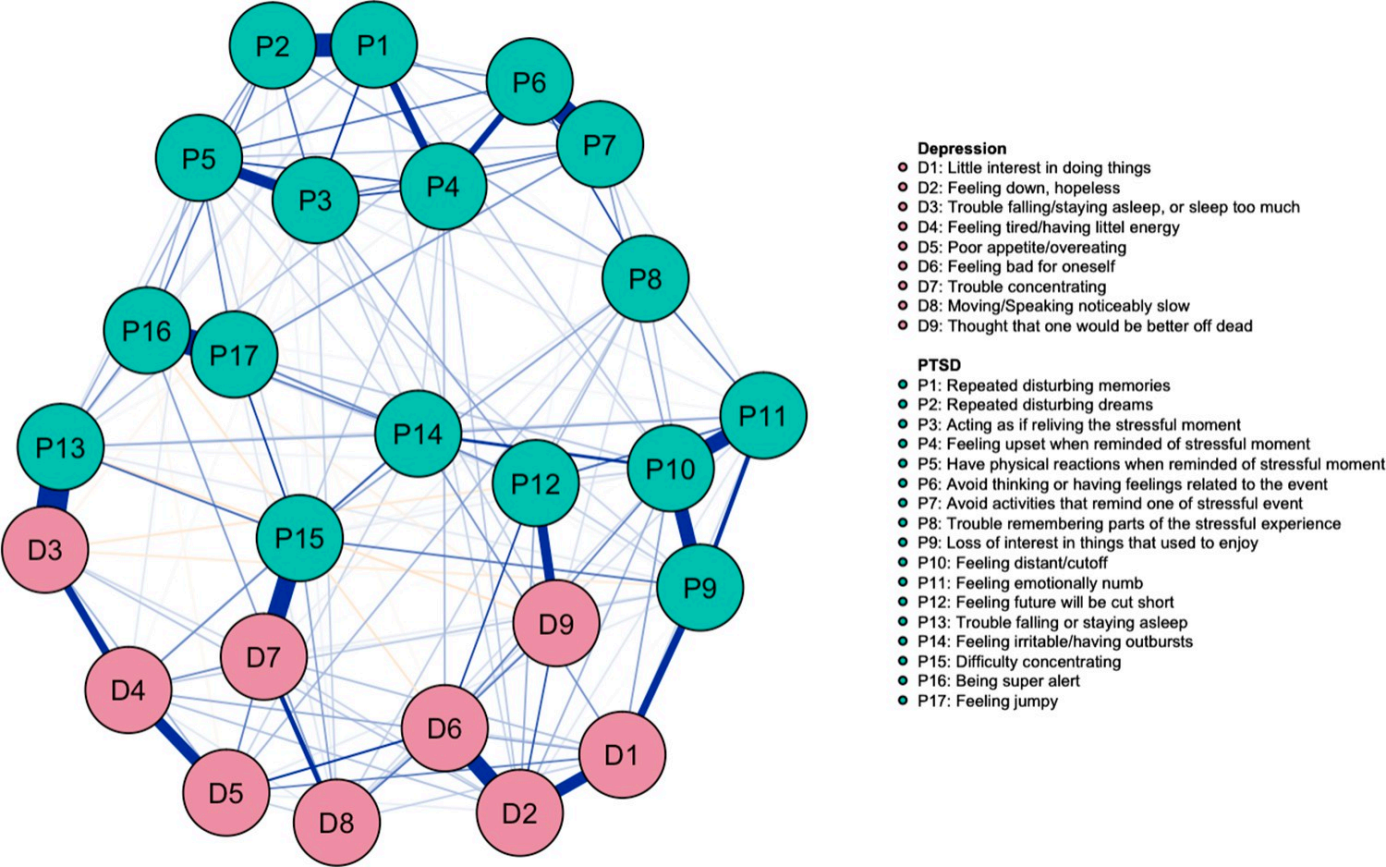
Network of PTSD and MDD symptoms structure for veterans with mTBI (N = 753). Positive relations are represented by blue edges and negative relations are represented by orange edges. The thicker and more saturated edges indicated stronger partial correlations between the nodes/symptoms.

The network of PTSD and MDD symptoms of the positive mTBI sample is depicted in Fig 1.

Overall, the strongest edges were within the PTSD symptoms cluster, and they were *trouble falling/staying asleep* (P13) -to- *trouble falling/staying asleep or sleep too much* (D3), *being super alert* (P16) -to- *feeling jumpy* (P17), *avoid thinking about the event* (P6) -to- *avoid activities that remind one of stressful event* (P7), *repeated disturbing memories* (P1) -to- *repeated disturbing dreams* (P2) and *feeling distant/cutoff* (P10) -to- *feeling emotionally numb* (P11). The strongest edges within MDD symptoms cluster were *feeling down/hopeless* (D2) -to- *feeling bad for oneself* (D6), and *little interest to doing things* (D1) -to- *feeling down/hopeless* (D2). The strongest edges across disorders were mainly overlapping symptoms: *trouble falling/staying asleep* (P13) -to- *trouble falling/staying asleep or sleep too much* (D3), *difficulty concentrating* (P15) -to- *trouble concentrating* (D7), *feeling future will be cut short* (P12) -to- *thought that one would be better off dead* (D9) and *loss of interest in things that used to enjoy* (P9) -to- *little interest in doing things* (D1). The tests of significance for edge weight difference are presented in S1 Fig in S1 File.

**Fig 2:**
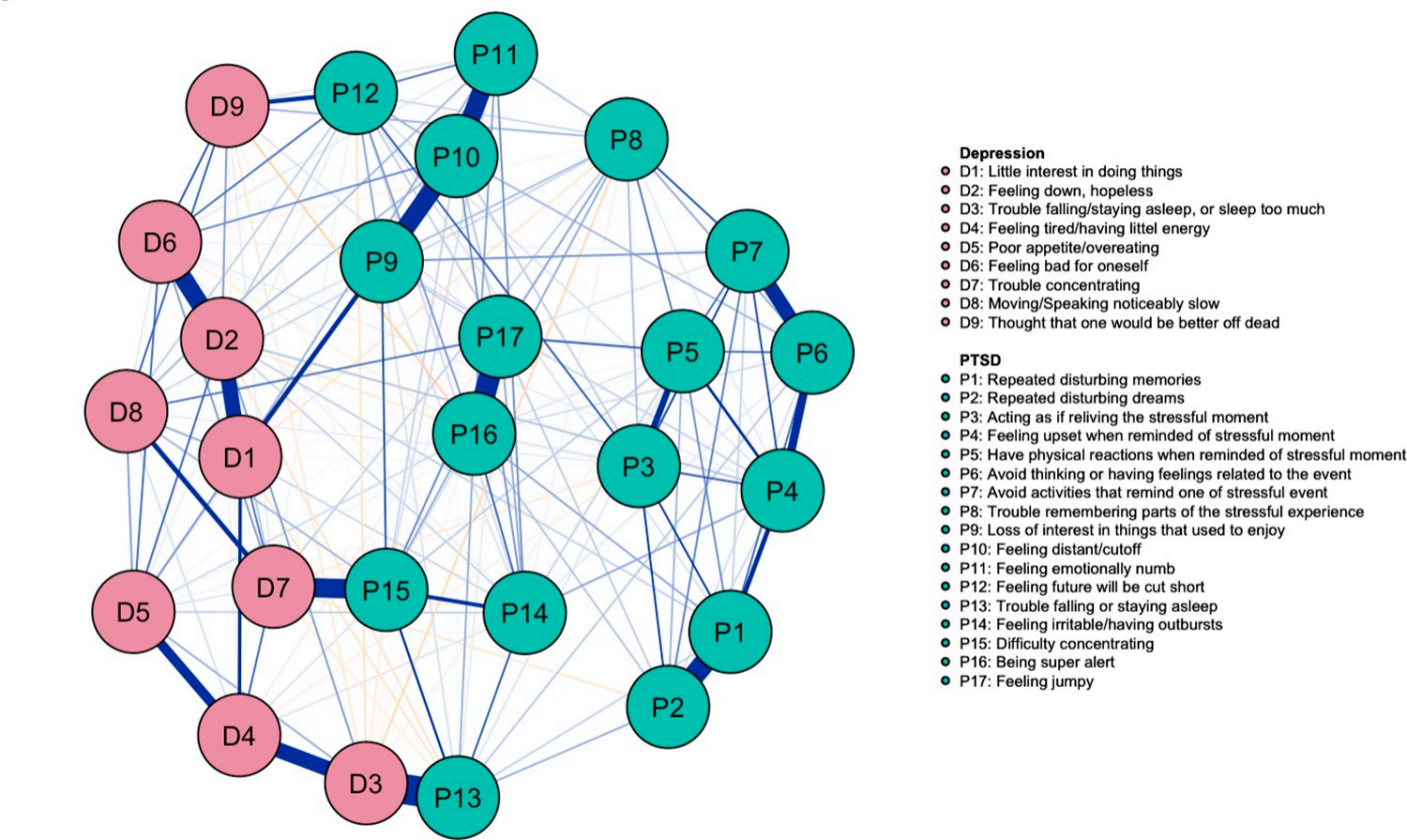
Network of PTSD and MDD symptoms structure for veterans without mTBI (N = 2044). Positive relations are represented by blue edges and negative relations are represented by orange edges. The thicker and more saturated edges indicated stronger partial correlations between the nodes/symptoms.

The network of PTSD and MDD symptoms of the negative mTBI sample is depicted in Fig 2.

Similar to the positive mTBI network, the overall strongest edges were within the PTSD symptoms cluster, and they were *trouble falling/staying asleep* (P13) -to- *trouble falling/staying asleep or sleep too much* (D3), *being super alert* (P16) -to- *feeling jumpy* (P17), *repeated disturbing memories* (P1) -to- *repeated disturbing dreams* (P2), and *feeling distant/cutoff* (P10) -to- *feeling emotionally numb* (P11). The strongest edges within MDD symptoms cluster were *feeling down/hopeless* (D2) -to- *feeling bad for oneself* (D6) and *trouble falling/staying asleep or sleep too much* (D3) -to- *feeling tired/having little energy* (D4). The strongest edges across disorders were again mainly overlapping symptoms: *trouble falling/staying asleep* (P13) -to- *trouble falling/staying asleep or sleep too much* (D3), *difficulty concentrating* (P15) -to- *trouble concentrating* (D7), *feeling future will be cut short* (P12) -to- *thought that one would be better off dead* (D9) and *loss of interest in things that used to enjoy* (P9) -to- *little interest in doing things* (D1). The tests of significance for edge weight differences are presented in S2 Fig in S1 File

The stability values of the estimated networks (CS-coefficients) were 0.75 for EI and network strength for both samples, indicating highly stable networks. EI appears to be a slightly more stable centrality measure than network strength, which justifies our choice of using EI to infer node/symptoms centrality/importance (See S3C & S3D Fig in S1 File). The non-parametric bootstrapped tests suggest moderately accurate estimations of edge weights (See S3A & S3B Fig in S1 File).

**Fig 3:**
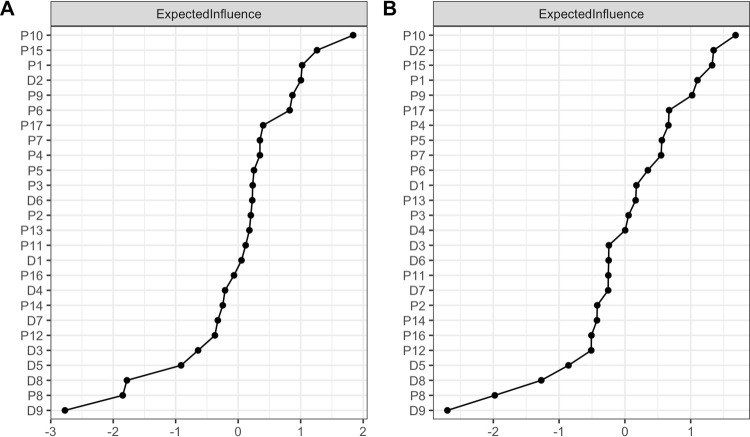
Network centrality–Expected influence. A. The EI measure for PTSD and MDD symptoms network among positive mTBI veterans. B. The EI measure for PTSD and MDD symptoms among negative mTBI veterans.

The standardized EIs for the two networks are depicted in Fig 3.

The most influential symptoms were *feeling distant/cutoff* (P10) and *difficulty concentrating* (P15) for veterans with mTBI and *feeling/distant/cutoff* (P10) and *feeling down/hopeless* (D2) for veterans without mTBI. For both groups, the least influential symptoms were *thought that one would be better off dead* (D9), *trouble remembering parts of the stressful event* (P8) and *moving/speaking noticeably slow* (D8). The tests of significance for node EI differences are presented in S4 and S5 Figs in S1 File. Centrality measures of node strength, closeness and betweenness are presented in S6 and S7 Figs in S1 File.

When considering only the bridge nodes, results were similar across the two networks. Results showed that the most influential bridge nodes were mostly the overlapping symptoms across the two disorders, they are *trouble falling/staying asleep* (P13), *difficulty concentrating* (P15) and *feeling future will be cut short* (P12) from PTSD symptoms cluster and *trouble falling/staying asleep or sleep too much* (D3), *trouble concentrating* (D7) and *little interest in doing things* (D1) from MDD symptoms cluster. The least influential bridge symptoms were different across the disorders, with *avoid thinking/having feelings related to the event* (P6) having the lowest EI for positive mTBI network and *being super alert* (P16) having the lowest EI for negative mTBI network (more details on S8 & S9 Figs in S1 File).

#### Network comparison tests

The positive mTBI and negative mTBI networks were similar in structure, with high correlation between the regularized symptoms interrelations of each group (r = 0.93). Moreover, the network structure invariance test was not significant (M = 0.14, permutations = 2000, p = 0.17), and the two networks did not differ significantly in regard to global network strength (S = 0.31, S_pos_ = 12.66, S_neg_ = 12.96, permutations = 2000, p = 0.43) and the network global EI (C = -0.03, permutations = 2000, p = 0.096).

#### MTBI network with clinical covariates

**Fig 4:**
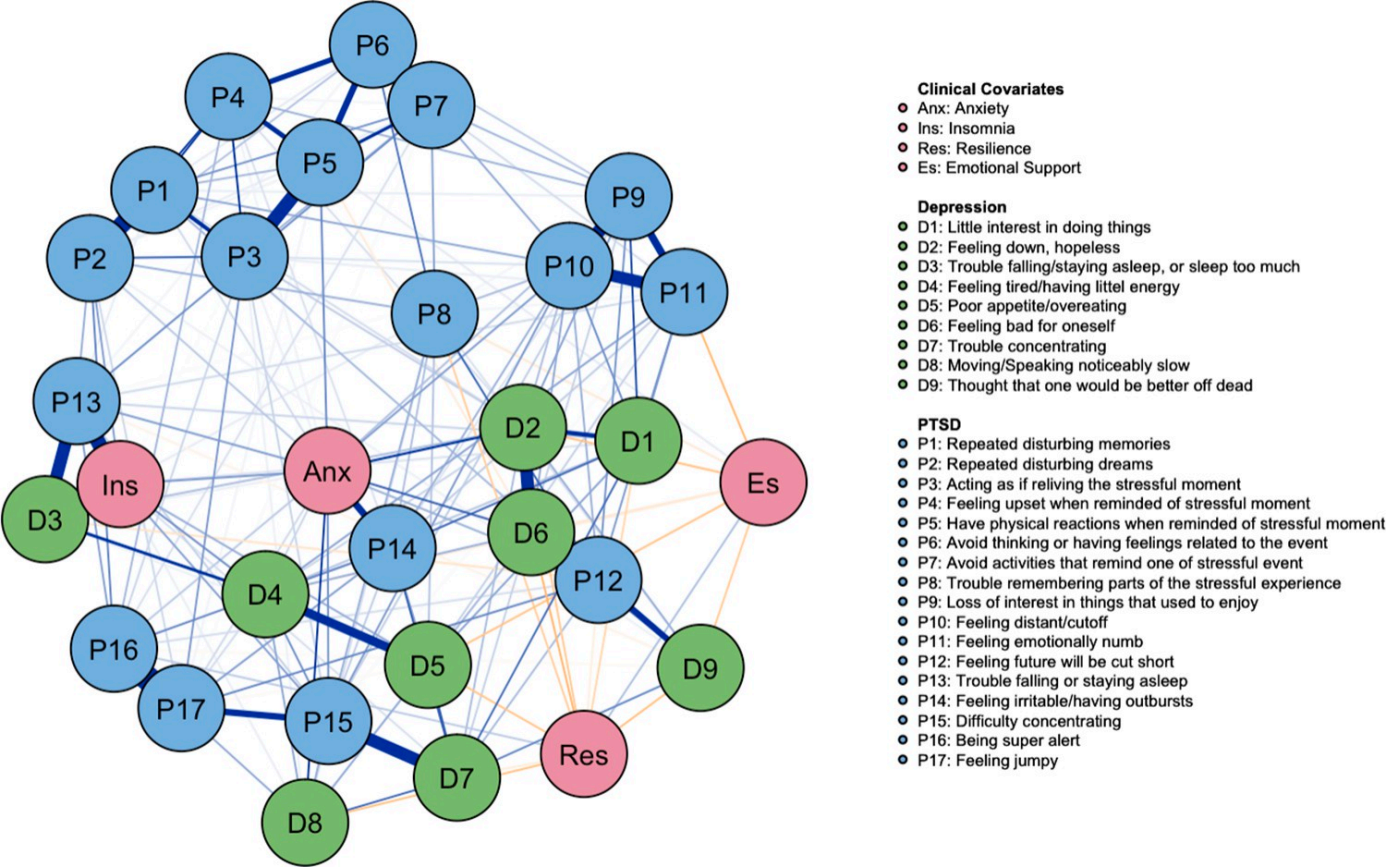
Network of PTSD and MDD symptoms with clinical covariates for veterans with mTBI (N = 234). Positive relations are represented by blue edges and negative relations are represented by orange edges. The thicker and more saturated edges indicated stronger partial correlations between the nodes/symptoms.

The network of PTSD and MDD symptoms with clinical covariates of a subset of the positive mTBI sample is depicted in Fig 4. The stability values of the estimated network (CS-coefficient) were 0.75 for EI and 0.67 for network strength, indicating highly stable networks. For edge weight accuracy, see S10 Fig in S1 File.

The strongest positive edges between the clinical covariates and the symptoms were *trouble falling/staying asleep* (P13) and *trouble falling/staying asleep or sleep too much* (D3) -to- *Insomnia score*, followed by *feeling irritable/having outbursts* (P14) -to- *Anxiety score*. The strongest negative edges between the clinical covariates were *feeling bad for oneself* (D6) -to- *Resilience score* and *feeling down/hopeless* (D2) -to- *Emotional Support score*. Specifically, most of MDD symptoms were negatively partially correlated with both the Resilience score and the Emotional Support score, while only *feeling emotionally numb* (P11) and *trouble remembering parts of the stressful event* (P8) of the PTSD symptoms were negatively correlated with Resilience score and Emotional Support score. The tests of significance for edge weight differences are presented in S11 Fig in S1 File.

**Fig 5:**
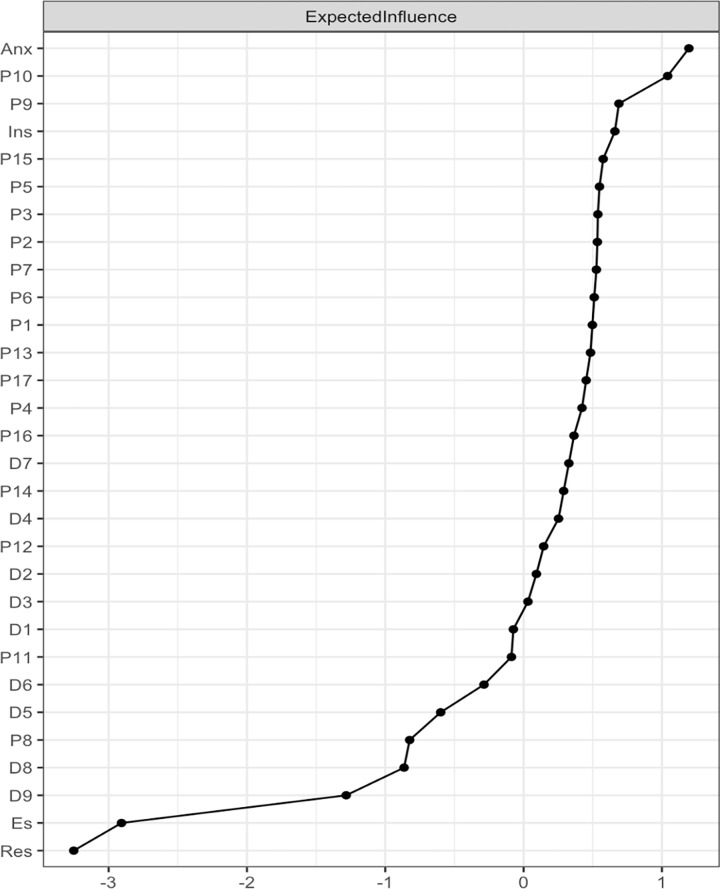
EI for positive mTBI PTSD & MDD symptoms with clinical covariates network.

Both Anxiety and Insomnia appeared to have high positive EI in the network, indicating their strong positive influence over PTSD and MDD symptoms. Moreover, both Resilience and Emotional Support appeared to have high negative EI in the network, indicating their strong negative influence over PTSD and MDD symptoms (as shown in Fig 5). The tests of significance for node EI differences are presented in S12 Fig in S1 File. Centrality measures of node strength, closeness and betweenness are presented in S13 Fig in S1 File.

## Discussion

This study examined the symptoms and comorbidity of PTSD and MDD across a sample of veterans with and without mTBI using the novel network approach. Past research suggests MDD and PTSD are among the two most common mental health sequalae occurring in mTBI populations and are strong predictors for persistent post-concussion symptoms and disability [8, 12, 30]. Results from our study supported this: PTSD and MDD are more prevalent and severe among veterans with mTBI than veterans without. Thus, it is important to proactively screen, monitor and provide treatment for PTSD and MDD symptoms post-mTBI. Unfortunately, one study found that less than half of patients with mental health complication(s) following injury were referred to appropriate care and received timely treatment [63]. Therefore, important gaps between knowledge and practice remain. One solution is to implement routine mental health screening, such as eScreening, in primary care settings. EScreening provides customized and automated self-report mental and physical health screening and has been proven to be an effective tool for initial and ongoing symptoms monitoring with high efficiency and patient satisfaction [48, 64]. Successful implementation of highly effective screening methods like eSreening in primary care settings could potentially improve the detection and monitoring of mental health symptoms post-MTBI and expedite the referring processes of veterans to adequate care.

To further assess the PTSD and MDD symptomology after mTBI, we examined the positive mTBI network structure of the PTSD/MDD symptoms. *Feeling distant/cutoff* (P10) and *difficulty concentrating* (P15) emerged as the two most central nodes in the positive mTBI network, echoing the findings of previous studies [41, 44, 65]. The centrality of these two symptoms suggested their importance within the PTSD and MDD network and their strong influence over other symptoms. Although further evaluation of the symptoms and its effect on the development of disorders is required, our results provide preliminary targets for screening, monitoring and treatment for post-mTBI PTSD and MDD. For example, it might be useful to develop brief screening measures that assess for emotional numbness and concentration problems post-mTBI to intervene early and prevent the development of other PTSD and MDD symptoms. The implementation of post-mTBI rehabilitation interventions that address concentration, emotional numbness and sleep problems etc., such as CogSMART, can also be an effective way to improve post-mTBI treatment outcomes [66, 67].

Moreover, when considering only the bridging nodes, overlapping symptoms between the disorders—sleep and concentration problems—came up as the most central nodes, which is consistent with literature showing overlapping symptoms are an origin for high comorbidity [44, 47, 68]. However, there is ongoing debate as to whether PTSD and MDD comorbidity is driven by overlapping symptoms or whether such symptoms constitute a subtype of PTSD [69]; thus further clarifying research is needed. Similar to previous research, *trouble remembering parts of the stressful event* (P8) were found to be among the least central nodes in the network [44, 46], suggesting that it might not be as important in PTSD and MDD detection and symptoms development.

We also examined the PTSD and MDD symptom structure network in veterans with and without mTBI. The central nodes and bridge nodes were similar across the networks, and there were no significant differences in network structure, global strength and global expected influence. The results suggested that although mTBI is indicative of higher likelihood to develop more severe PTSD and/or MDD, mTBI does not seem to significantly influence the symptoms structure of co-morbid PTSD and MDD. Since no studies have investigated the PTSD and MDD symptom structure post-mTBI, our findings provide preliminary knowledge on which future studies should build.

Sleep symptoms across PTSD and MDD consistently appeared as the strongest bridge nodes, and the edge connecting two sleep symptoms/nodes were the strongest across the two networks. Our results are consistent with previous network studies that found *sleep difficulty* to be the major bridging nodes across various populations [46, 47]. This is not surprising as insomnia/sleep problems are a risk factor for many mental health problems, such as depression and PTSD [70, 71]. Insomnia is also related to worse self-rated health among older veterans [72]. Thus, symptoms of sleep difficulty within PTSD and MDD appear to be an important treatment target for post-mTBI care; and providing sleep-related interventions like Cognitive Behavioral Therapy-Insomnia may be highly beneficial for this population (i.e., veterans with mTBI) [73–75].

Last but not the least, we examined a network of PTSD and MDD symptoms with several clinical covariates to understand how these added variables influence the network structure. We found that both anxiety and insomnia have high positive EI within the network, that is, anxiety and insomnia are highly influential over PTSD and MDD symptoms. Additionally, the edges between PTSD/MDD symptoms such as sleep problems to insomnia, and irritability to anxiety were among the strongest. Previous research has suggested that insomnia is a strong predictor rather than outcome for PTSD and MDD symptom development among veterans [71], which is consistent with our findings. Anxiety has also been shown to predict the deterioration of PTSD symptoms [76], which is similar to our results that showed a strong link between anxiety and PTSD symptoms. Thus, in addition to MDD and PTSD, it is important to routinely screen for and monitor anxiety and insomnia to improve the quality and effectiveness of post-mTBI care.

Emotional support and resilience also came up as influential factors in the positive mTBI with clinical covariates network, such that they demonstrated high negative EIs in the network and were negatively linked to most of the MDD symptoms and some of the PTSD symptoms. The results suggest that emotional support (or perceived social support) and resilience both appear to be strong protective factors for PTSD and MDD. Previous research has shown that perceived social support is associated with better mental health outcomes and reduced symptoms severity [77, 78]. Moreover, resilience has been found to be a promising buffer for mental health, physical health, and well-being [79–81]. Interventions promoting resilience have been associated with reductions in stress and depression [82], but such resilience building practices have not been widely adopted in large healthcare organizations [83]. VA is working to improve quality of care by shifting from a reactive, disease-focused care model to a proactive, patient-centered approach, which aims to develop a personalized health plan based on the unique values, needs, and goals (e.g., building resilience, improve emotional support) of every individual [84, 85]. Therefore, systematically develop and evaluate interventions that incorporate techniques which enhance patients’ perception of emotional support, or interventions that promotes resilience in the context of post-mTBI care align with VA’s focus on this personalized health approach.

The study has a number of limitations. As eScreening was originally developed for Transition Care Management programs, which coordinate health care for post-9/11 veterans at the point of enrollment, the data available for this study were limited to post-9/11 era veterans. The current sample consist of mostly male (84.7%), caucasian (59.8%), Navy (48.8%) or Marines (32.1%) veterans. Thus, findings may not generalize to the larger veteran population. Second, the data for this study is cross-sectional, which limits our ability to make temporal inferences and to draw directions between the symptoms [86]. Third, the network analysis approach assumes that all relative and influential variables are incorporated in the network, but we may have missed some important symptoms and/or non-symptoms that play an important role in the comorbidity network. Although we tried to address this problem by including clinical covariates, there still exist many other clinical symptoms/comorbidities (e.g., substance use, physical disability) [25, 87, 88] and/or factors (e.g., coping styles, employment status, stressful life events and combat exposure) [42, 89–91] that might be vital in the PTSD and MDD network. Fourth, the study used data from service-seeking veterans registering for care through the VASDHS, of which the veterans reported various degrees of symptoms that may affect the network accuracy. The veterans reported symptoms severity ranged from sub-threshold PTSD/MDD to severe PTSD/MDD in our samples, while previous study suggested that sub-threshold PTSD symptoms structure might be different from moderate to severe PTSD symptoms structure [46]. Fifth, although the positive mTBI with clinical covariates network showed promising stability and accuracy, the sample for this network is still relatively small. The use of a larger sample would significantly improve accuracy and stability of the network, and thus allow us to make stronger inferences. Finally, the BTBIS used in the current study is a short screening tool for probable mTBI. We might overestimate the actual prevalence rate of mTBI among veterans using BTBIS. Future study should use a more valid measure for mTBI (e.g., clinician diagnoses based on standard criteria) to improve the validity of the results.

Despite these limitations, the current study has many strengths and can contribute to extant knowledge in the field. The study provides quantitative evidence for the greater prevalence and severity of PTSD and MDD symptoms and comorbidity after mTBI. The study also is the first and largest to examine the comorbid PTSD and MDD symptom structure in a sample of veterans with mTBI; and identified *Feeling distant/cutoff* (P10) and *difficulty concentrating* (P15) as the most central symptoms in the network and sleep problems as the most prominent bridge nodes across the disorders. These symptoms might be particular useful targets for screening, monitoring and treatment planning for post-MTBI mental health care and for improving treatment outcomes. Further, the use of effective screening tools, like eScreening, could potentially improve the detection of mental health symptoms post-mTBI and expedite the referral to appropriate care [48, 64]. Future research should use longitudinal data to better inform the development of post-mTBI comorbid PTSD and MDD symptoms; future research should also incorporate more potential influential factors for the progression or prevention of PTSD and MDD symptoms and examine the network structure in a larger veteran sample.

## Supporting information

S1 File S1 Table and S1–S13 Figs. (DOCX)

S1 Data (XLSX)
