# Supplementary material for: Symptoms of major depressive disorder and post-traumatic stress disorder in veterans with mild traumatic brain injury: A network analysis
Source: PLoS One. 2023 May 4;18(5):e0283101. doi: 10.1371/journal.pone.0283101 (PMC10159137; doi:10.1371/journal.pone.0283101)
Supplement: S1 File — (DOCX) [file pone.0283101.s001.docx]

**Supporting Information**

**Table S1. Chi Square test statistics for rates of PTSD, MDD and Comorbidity across mTBI and non-mTBI samples**

|  | Positive mTBI  *N (%)* | Negative mTBI  *N (%)* | *Chi Square* | *P* |
| --- | --- | --- | --- | --- |
|  |  |  |  |  |
| PTSD | 563 (74.8) | 871 (42.6) |  |  |
| Non-PTSD | 190 (25.2) | 1173 (57.4) | 227.73*** | <0.001 |
| MDD | 416 (55.3) | 659 (32.24) |  |  |
| Non-MDD | 337 (44.7) | 1385 (67.8) | 123.08*** | <0.001 |
| Comorbidity | 406 (53.9) | 582 (28.5) |  |  |
| Non-Comorbidity | 347 (46.1) | 1462 (71.5) | 155.94*** | <0.001 |

Note: PTSD is determined by a clinically cutoff score of 30 on the PCL-C and MDD is determined by a score of 10 on the PHQ-9. The frequency refers to rate of each mental health problem or non-mental health problem under either positive or negative mTBI (e.g., PTSD under positive mTBI = 74.8% and non-PTSD under positive mTBI = 25.2%, which adds up to 100%).

**
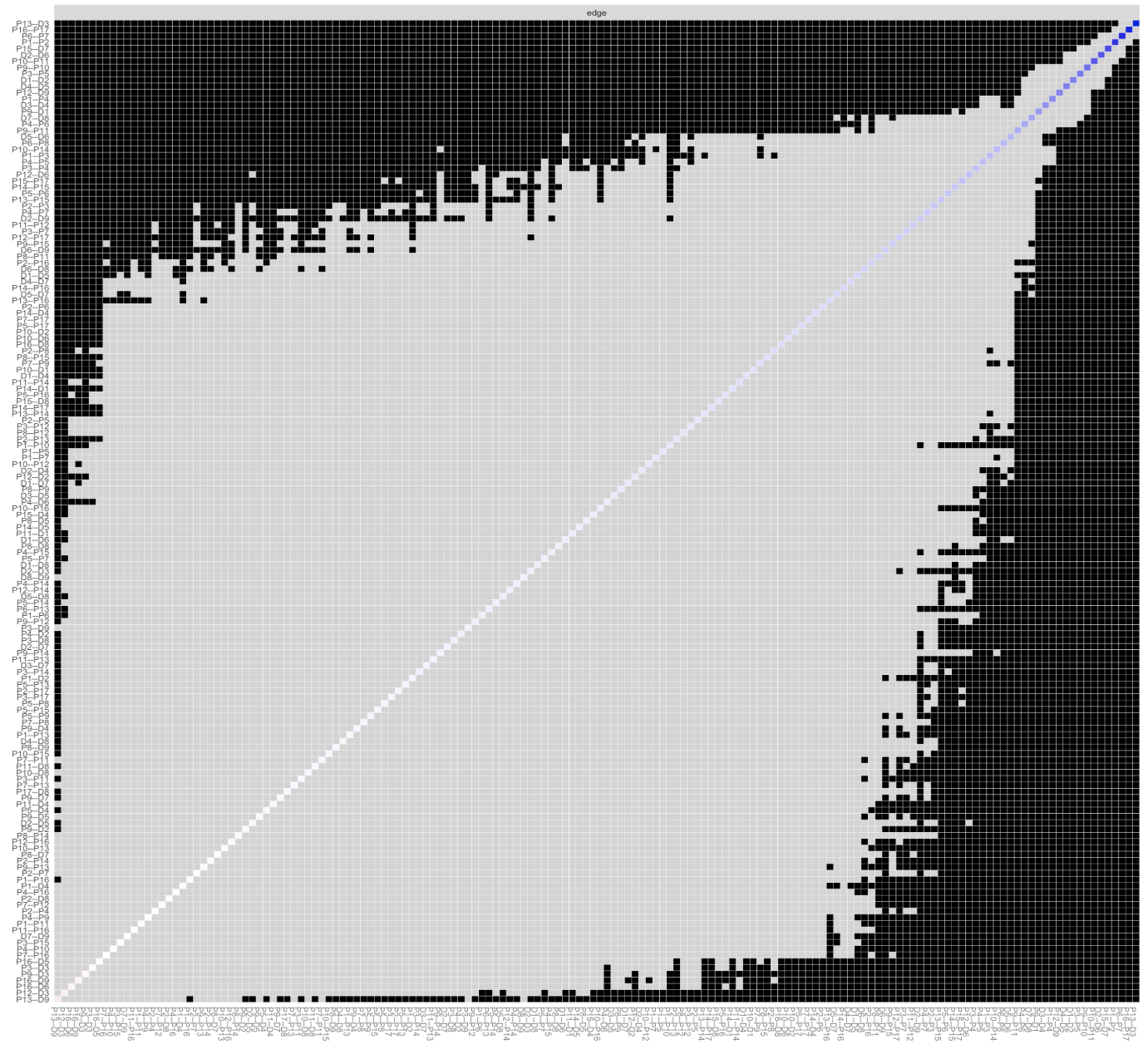
**

**Figure S1**. Bootstrapped difference tests (α = 0.05) between edge-weights for PTSD/MDD symptoms network among 753 veterans with MTBI. Black boxes represent edges that differ significantly from other edges and gray boxes represent edges that do not differ significantly from each other. Colored boxes correspond to the color of the edge in Figure 1.


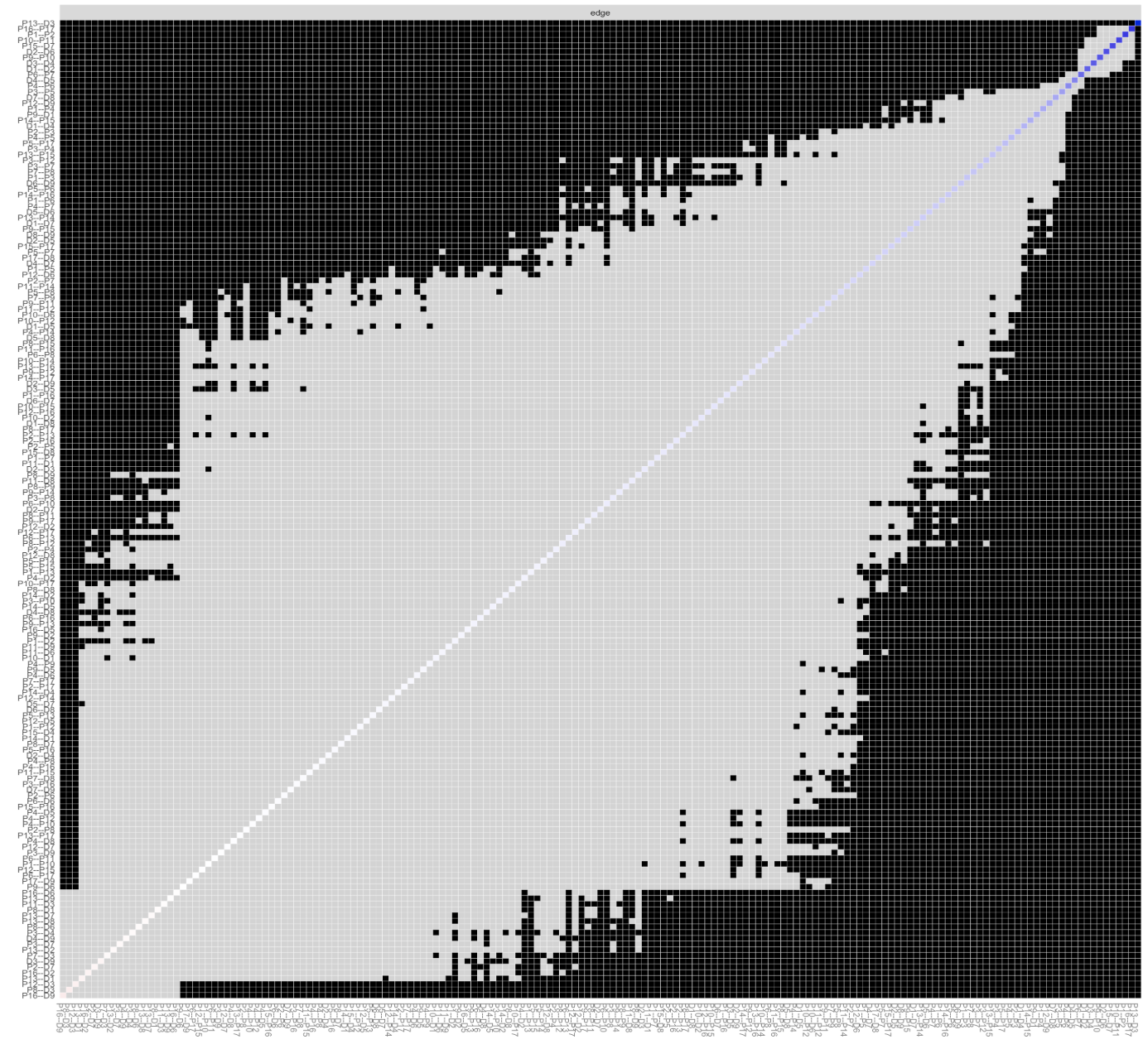


**Figure S2**. Bootstrapped difference tests (α = 0.05) between edge-weights for PTSD/MDD symptoms network among 2044 veterans without MTBI. Black boxes represent edges that differ significantly from other edges and gray boxes represent edges that do not differ significantly from each other. Colored boxes correspond to the color of the edge in Figure 2.

*
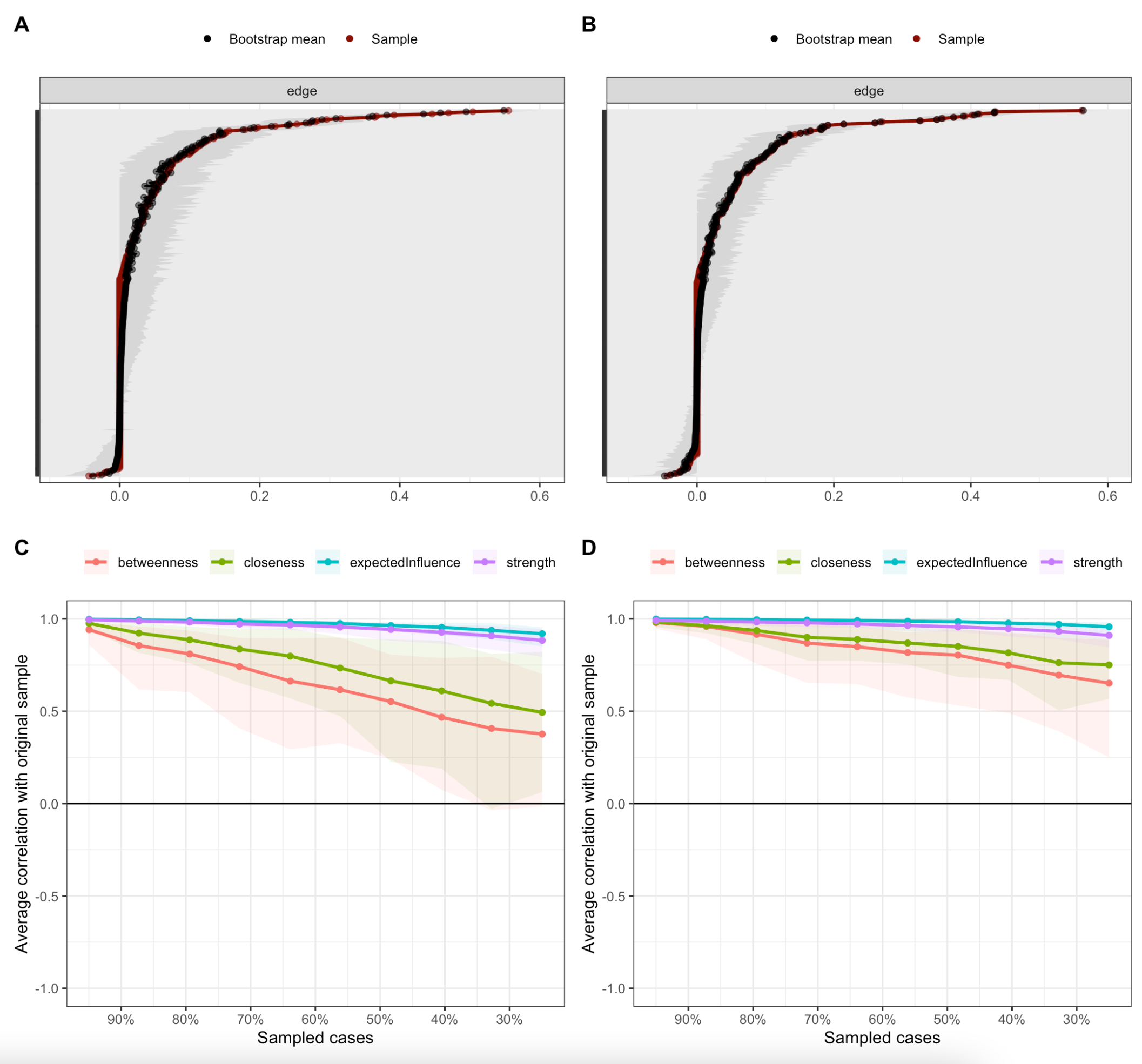
*

**Figure S3.** Network Robustness Measures. A. Bootstrapped 95% confidence interval of all edge weights of PTSD and MDD symptoms network among veterans with mTBI. B. Bootstrapped 95% confidence interval of all edge weights of PTSD and MDD symptoms network among veterans without mTBI. For A and B: Sample values are indicated by the red line; the bootstrapped means are represented by the black dots; and the grey area indicates the bootstrapped 95% confidence intervals. Each horizontal line represents one edge, and the graph is ordered by the edge weight from highest to lowest. C. Bootstrapped node EI, strength, closeness and betweenness of PTSD and MDD symptoms network among veterans with mTBI. D. Bootstrapped node EI, strength, closeness and betweenness of PTSD and MDD symptoms network among veterans without mTBI.


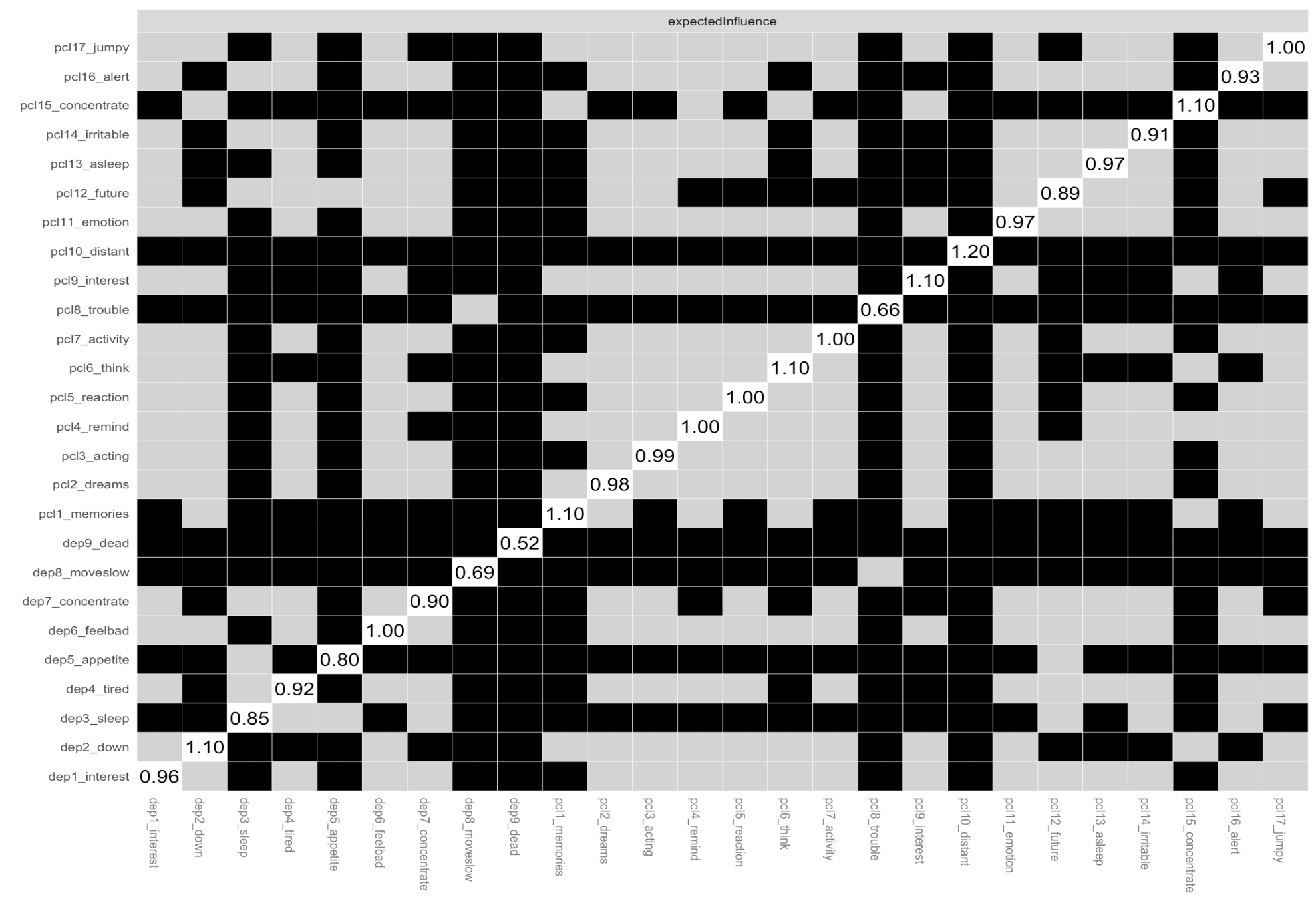


**Figure S4.** Bootstrapped difference tests (α = 0.05) between EI of the nodes in the PTSD/MDD symptoms network among 753 veterans with MTBI. Black boxes represent EI of the nodes that differ significantly from each other, and gray boxes represent EI of the nodes that do not differ significantly from other nodes.


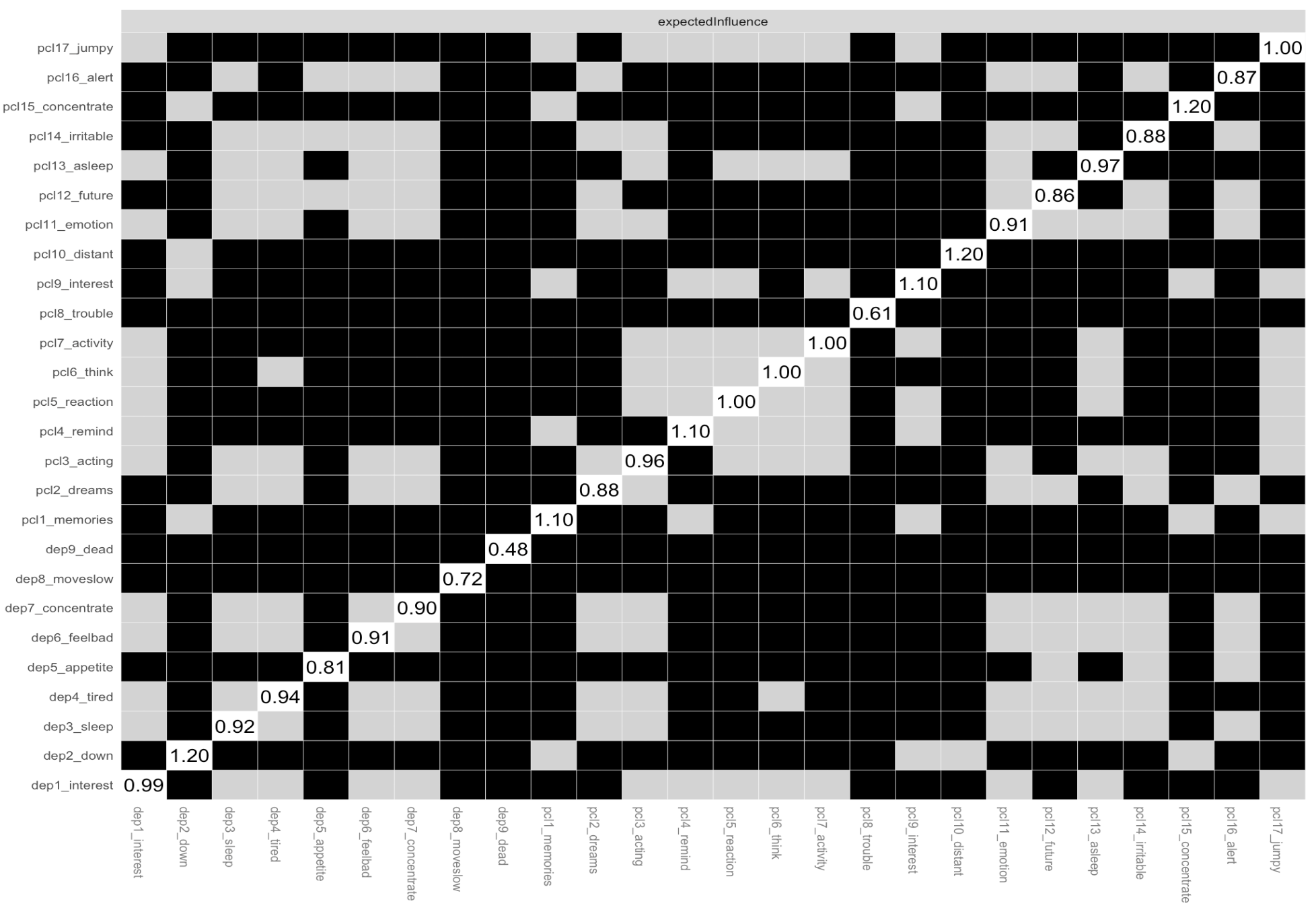


**Figure S5.** Bootstrapped difference tests (α = 0.05) between EI of the nodes in the PTSD/MDD symptoms network among 2044 veterans without MTBI. Black boxes represent EI of the nodes that differ significantly from each other, and gray boxes represent EI of the nodes that do not differ significantly from other nodes.


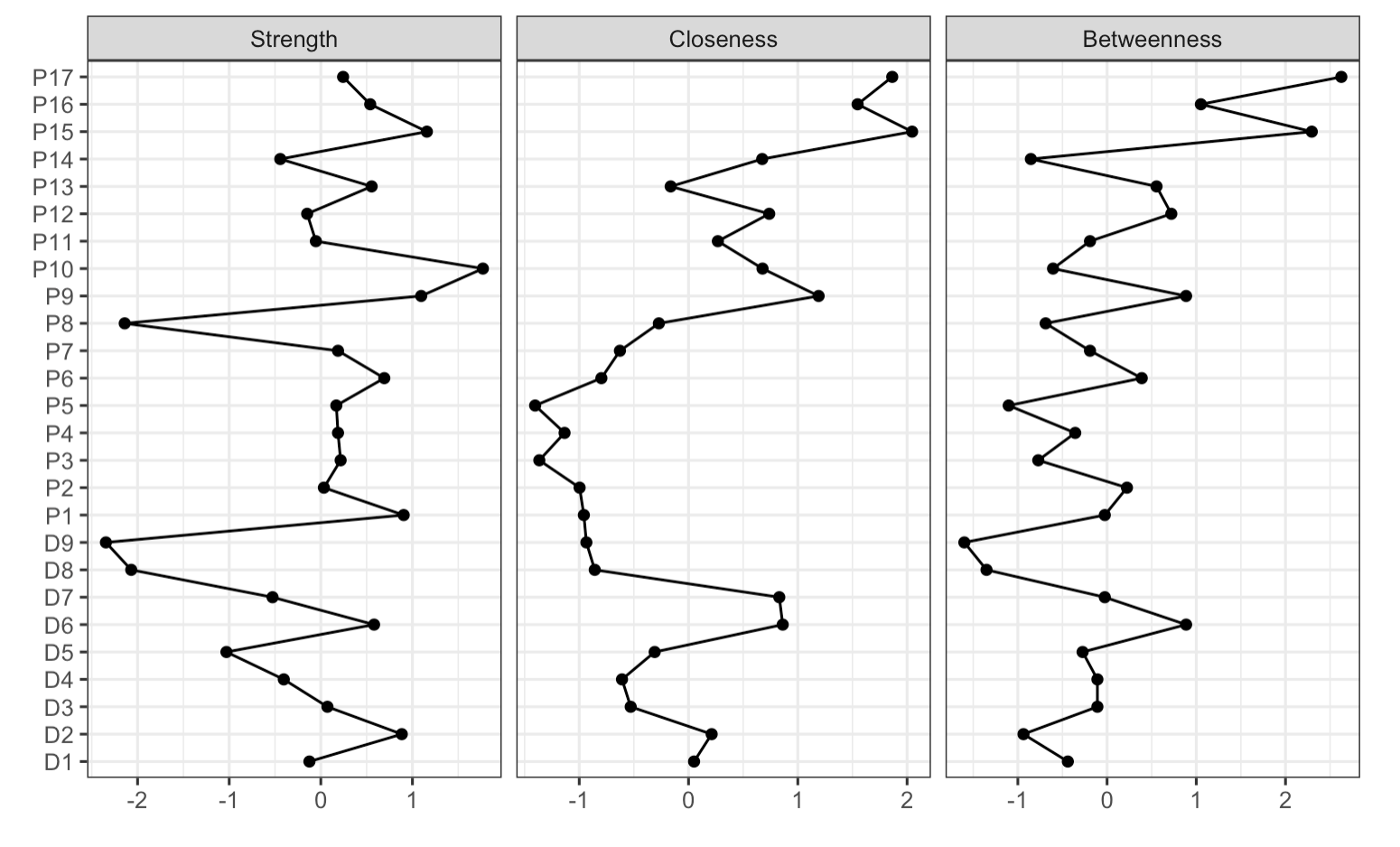


**Figure S6.** The strength, closeness and betweenness measure for PTSD and MDD symptoms network among positive MTBI veterans.


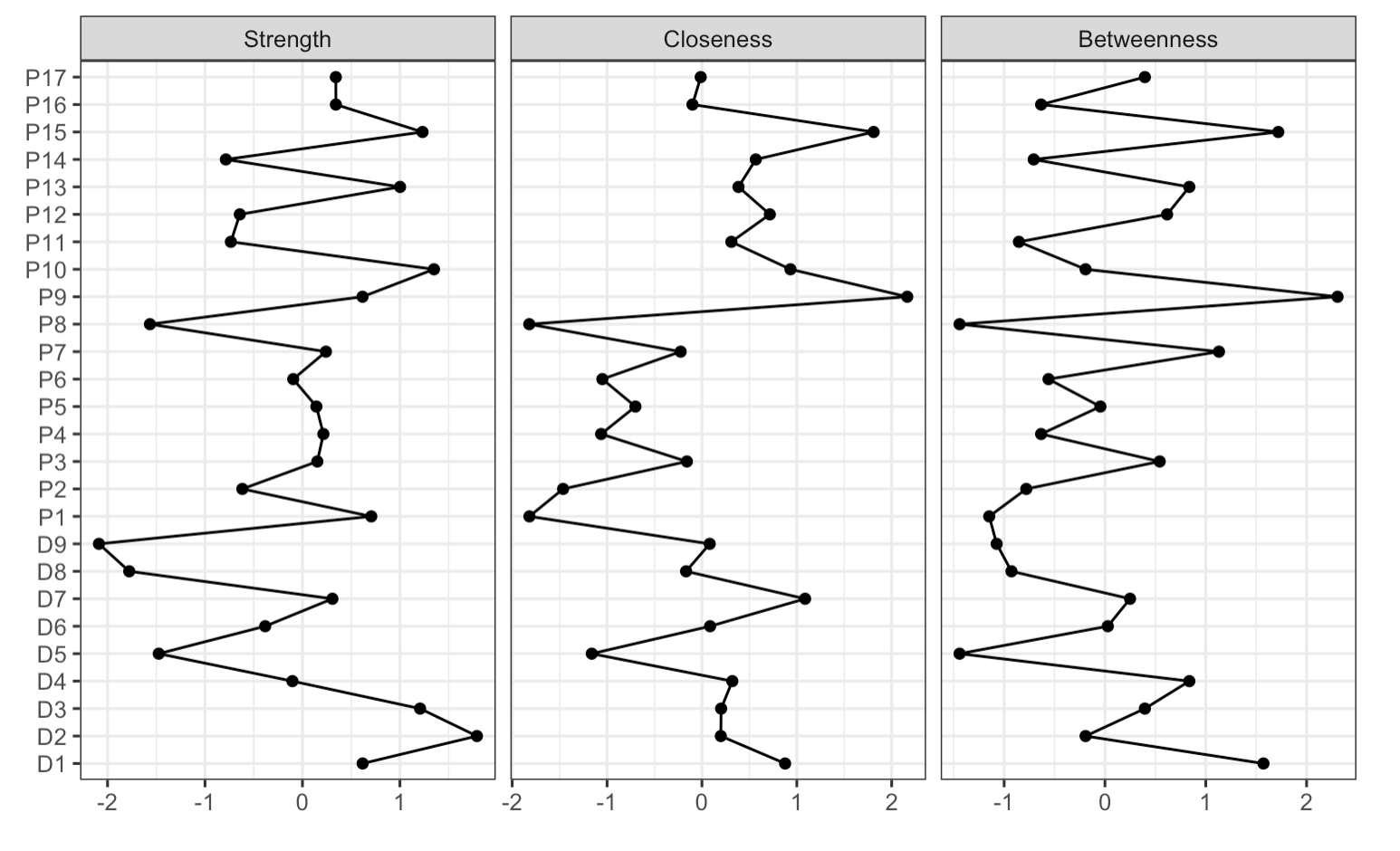


**Figure S7.** The strength, closeness and betweenness measure for PTSD and MDD symptoms network among negative MTBI veterans.


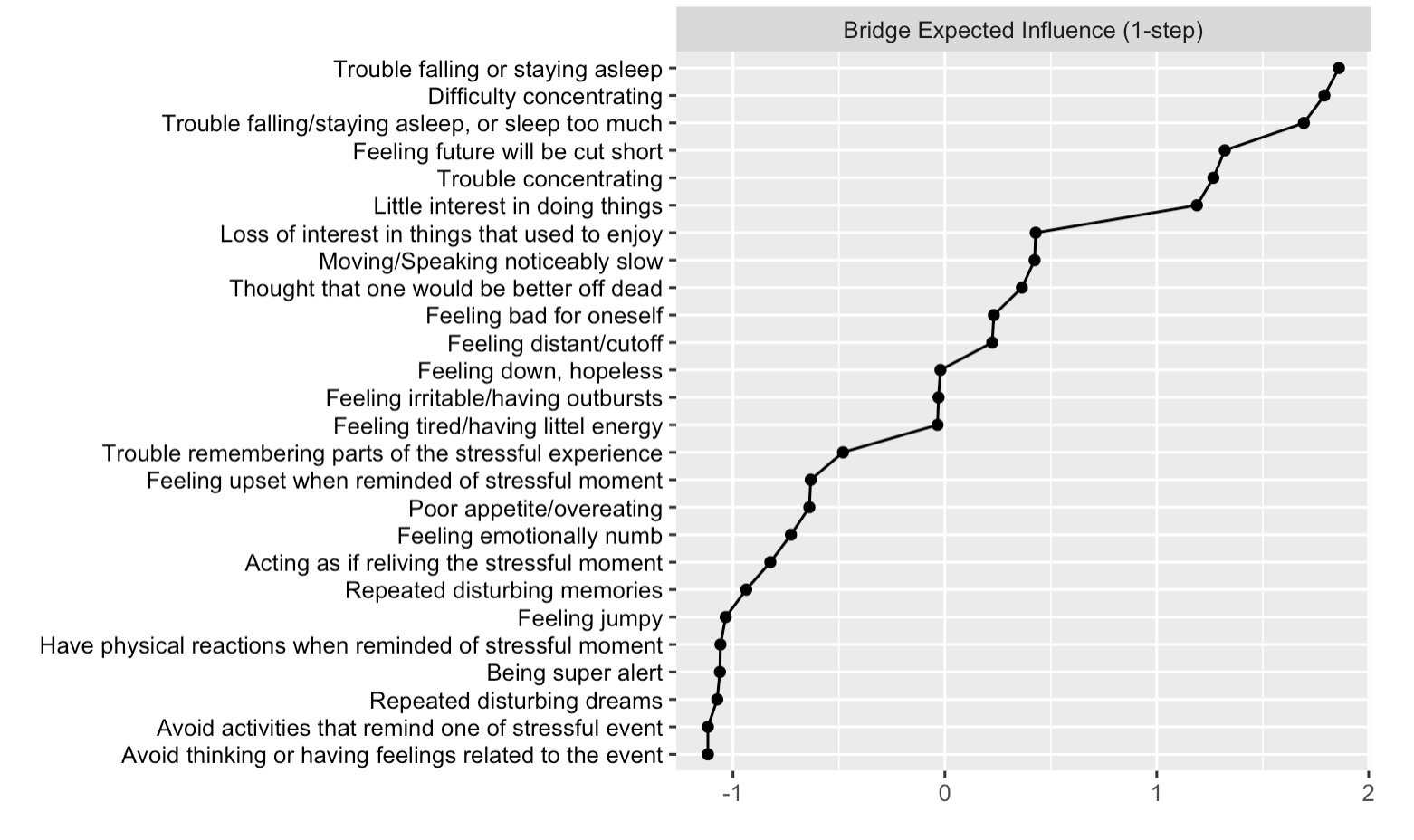


**Figure S8.** Bridge EI estimates for the PTSD/MDD symptoms network for 753 veterans with MTBI.


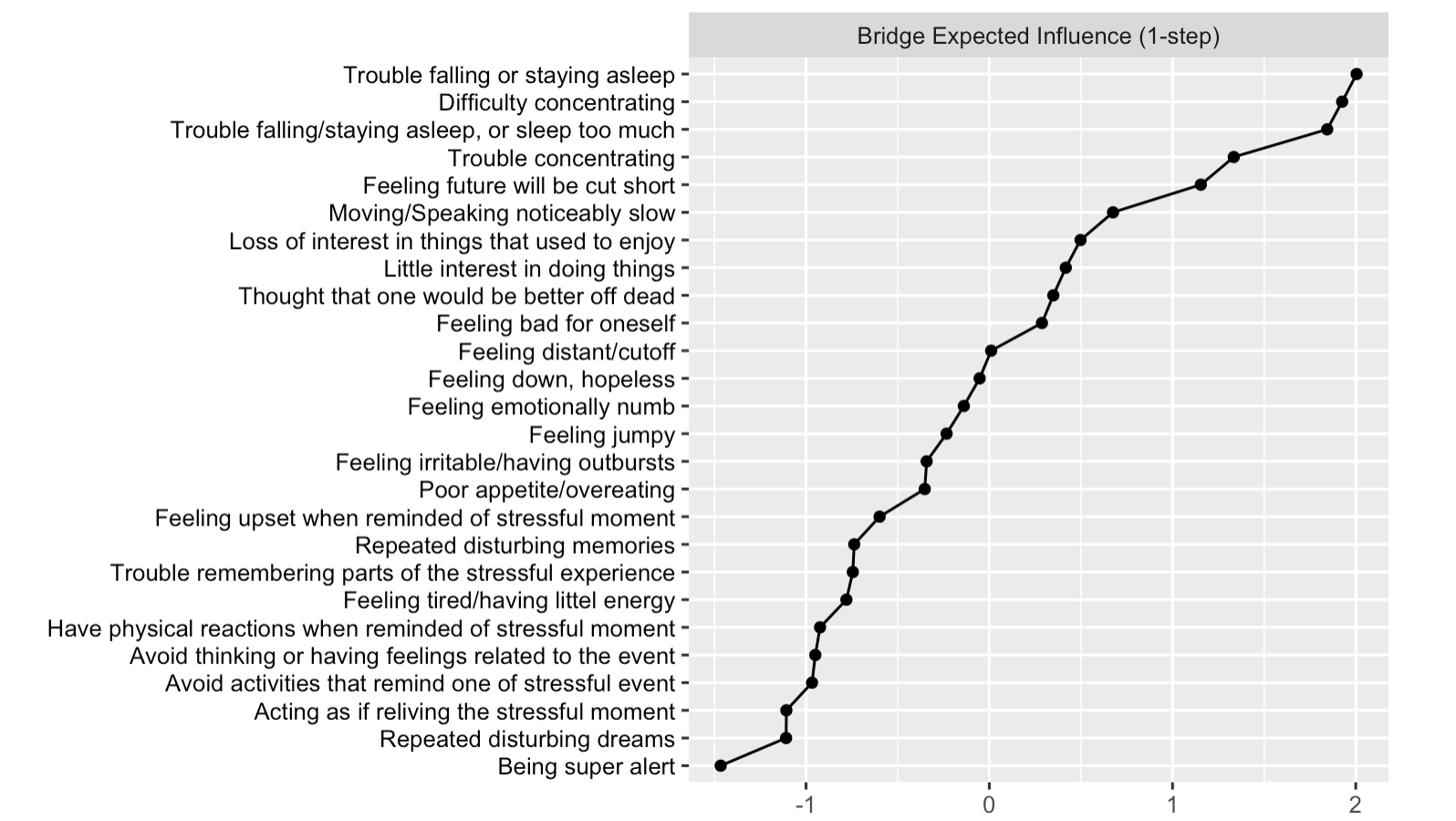


**Figure S9.** Bridge EI estimates for the PTSD/MDD symptoms network for 2044 veterans without MTBI.


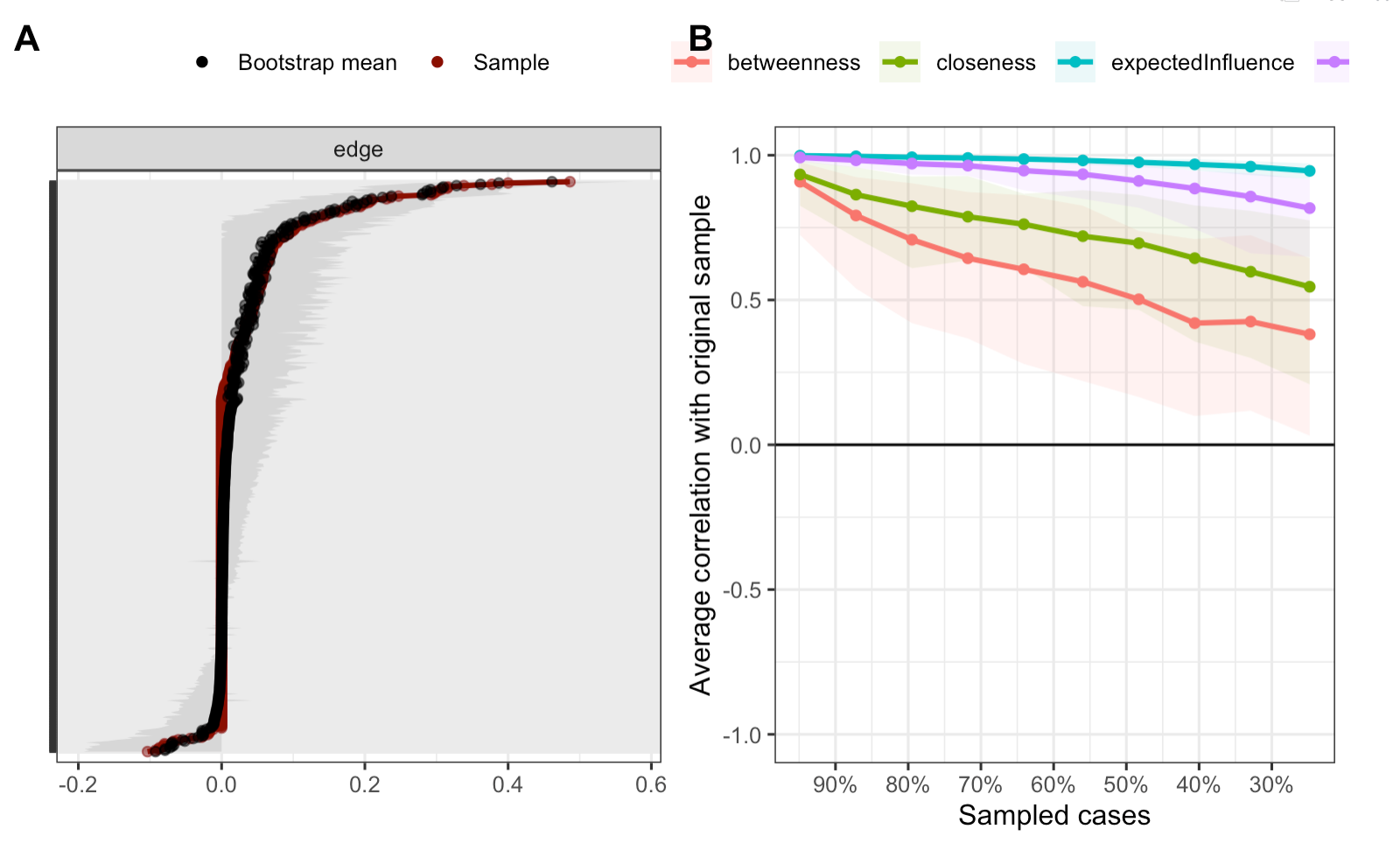


**Figure S10.** Network Robustness Measures. A. Bootstrapped 95% confidence interval of all edge weights of PTSD and Depression symptoms with clinical covariates network among veterans with MTBI. Sample values are indicated by the red line; the bootstrapped means are represented by the black dots; and the grey area indicates the bootstrapped 95% confidence intervals. Each horizontal line represents one edge, and the graph is ordered by the edge weight from highest to lowest. B. Bootstrapped node EI, strength, closeness and betweenness of PTSD and Depression symptoms with clinical covariates network among veterans with MTBI.


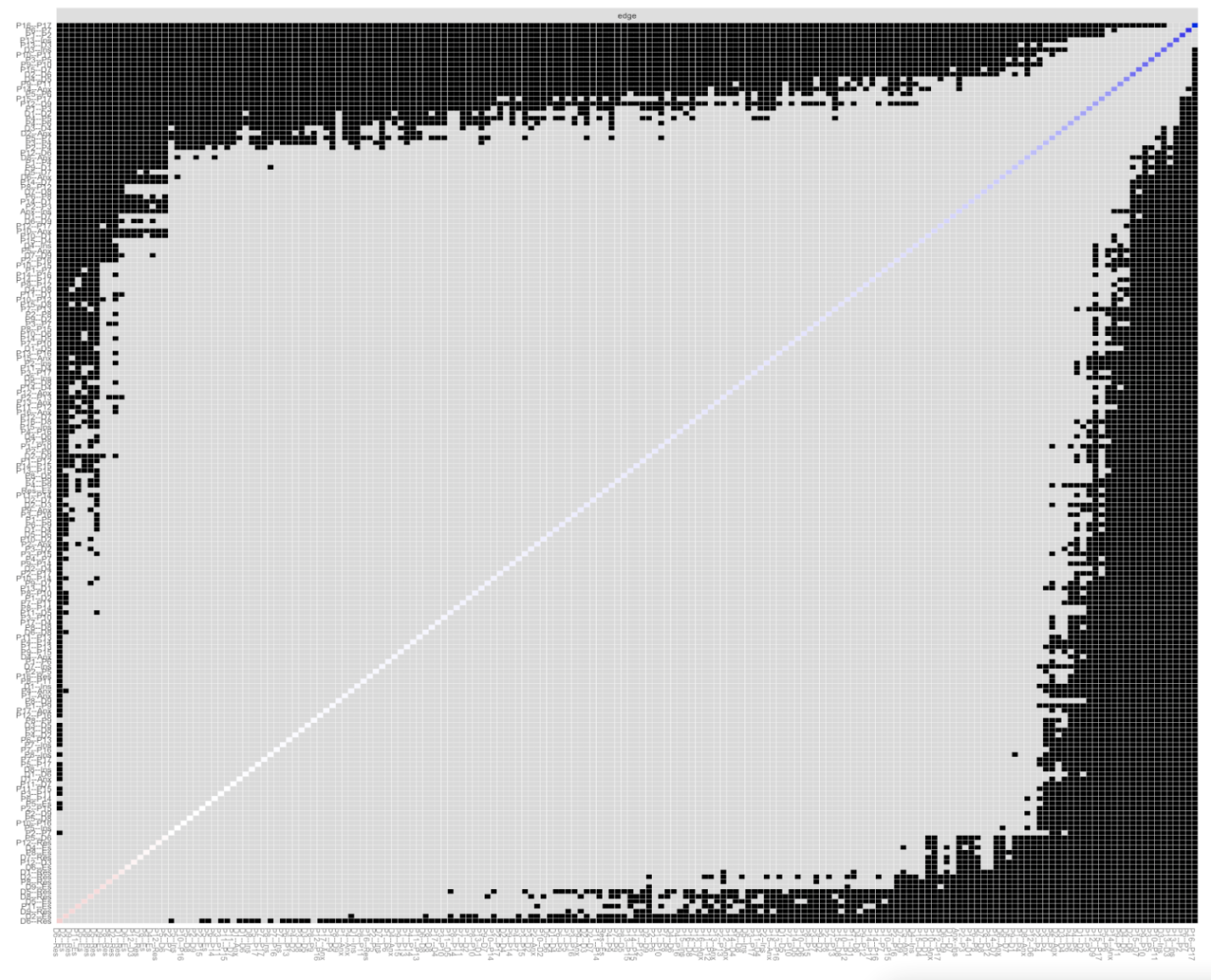


**Figure S11**. Bootstrapped difference tests (α = 0.05) between edge-weights for PTSD/MDD symptoms with clinical covariates network among 234 veterans with MTBI. Black boxes represent edges that differ significantly from other edges and gray boxes represent edges that do not differ significantly from each other. Colored boxes correspond to the color of the edge in Figure 5.


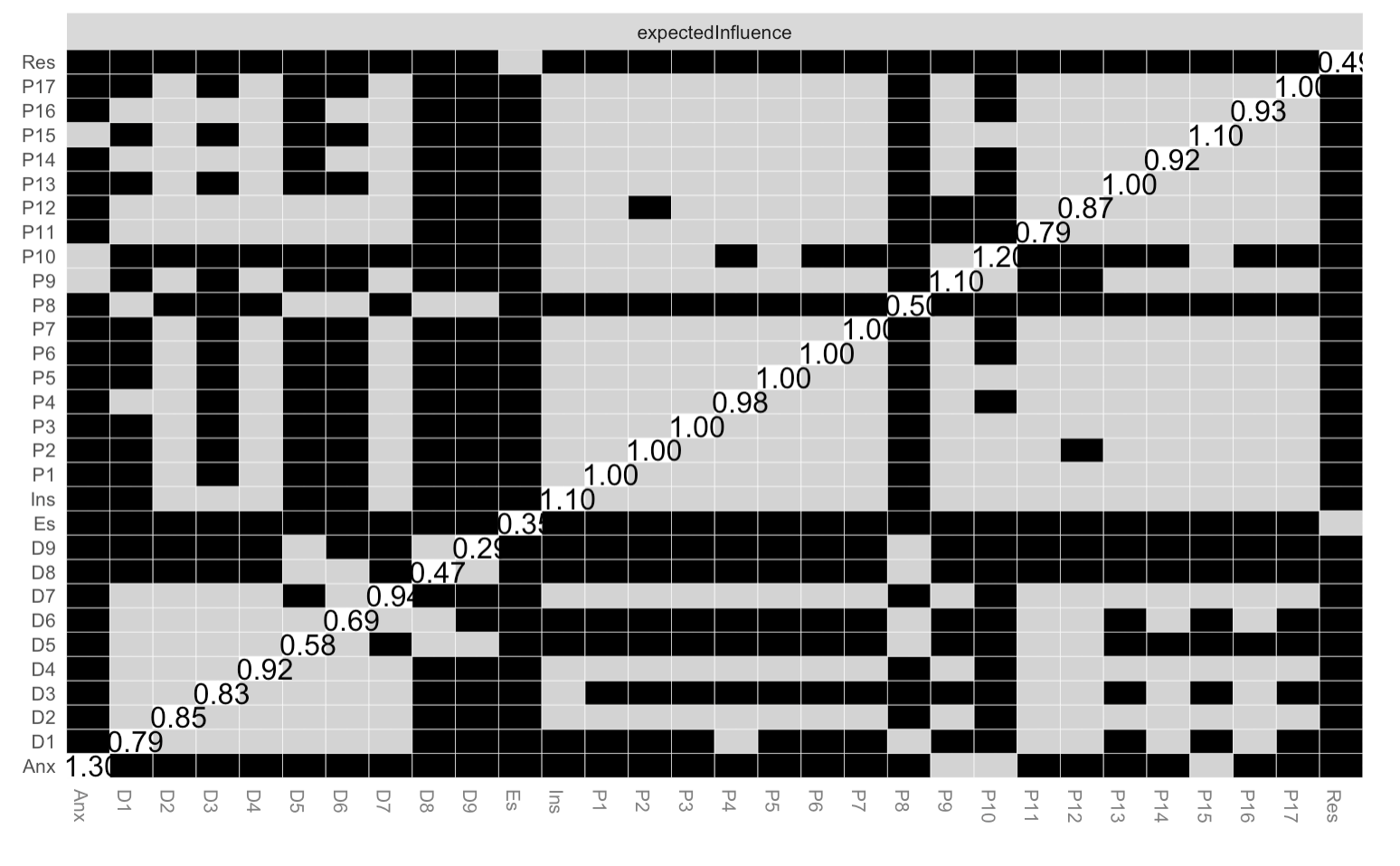


**Figure S12.** Bootstrapped difference tests (α = 0.05) between EI of the nodes in the PTSD/MDD symptoms with clinical covariates network among 234 veterans with MTBI. Black boxes represent EI of the nodes that differ significantly from each other, and gray boxes represent EI of the nodes that do not differ significantly from other nodes.


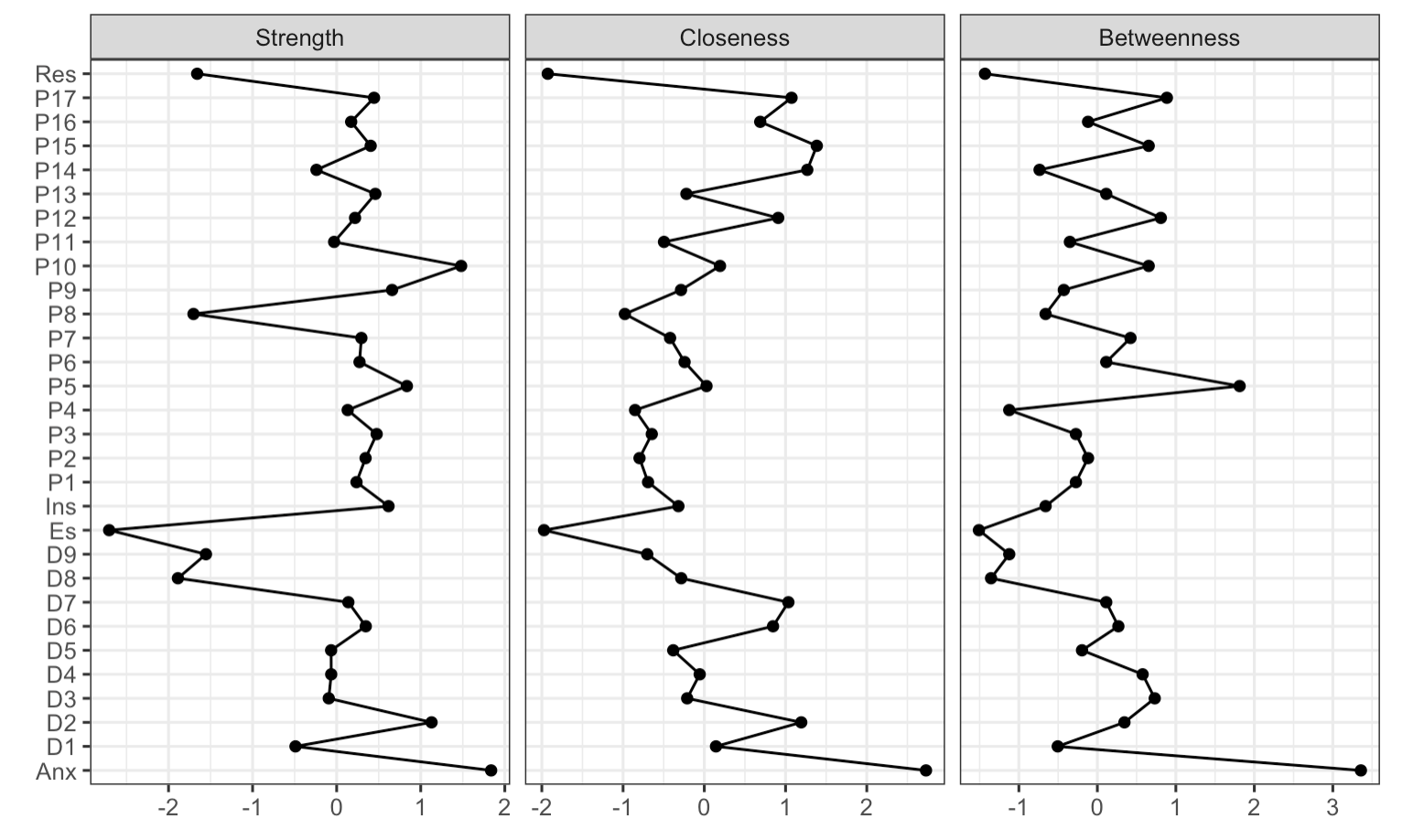


**Figure S13.** The strength, closeness and betweenness measure for PTSD and MDD symptoms with clinical covariates network among positive MTBI veterans.
